# Antioxidant evaluation-guided chemical profiling and structure-activity analysis of leaf extracts from five trees in *Broussonetia* and *Morus* (Moraceae)

**DOI:** 10.1038/s41598-020-61709-5

**Published:** 2020-03-16

**Authors:** Xinxin Cao, Lingguang Yang, Qiang Xue, Fan Yao, Jing Sun, Fuyu Yang, Yujun Liu

**Affiliations:** 1 National Engineering Laboratory for Tree Breeding, College of Biological Sciences and Biotechnology, Beijing Forestry University, Beijing, 100083 China; 2 College of Grassland Science and Technology, China Agricultural University, Beijing, 100193 China

**Keywords:** Chemical biology, Secondary metabolism

## Abstract

*Morus* and *Broussonetia* trees are widely used as food and/or feed. Among 23 phenolics identified from leaves of five Moraceae species using UPLC–QTOF–MS/MS, 15 were screened using DPPH/ABTS-guided HPLCs, including seven weak (flavonoids with one hydroxyl on B-ring) and eight strong (four caffeoylquinic acids and four flavonoids, each with a double hydroxyl on B-ring) antioxidants. We then determined the activity and synergistic effects of individual antioxidants and a mixture of the eight strongest antioxidants using DPPH-guided HPLC. Our findings revealed that (1) flavonoid glucuronide may have a more negative effect on antioxidant activity than glucoside, and (2) other compounds in the mixture may exert a negative synergistic effect on antioxidant activity of the four flavonoids with B-ring double hydroxyls but not the four caffeoylquinic acids. In conclusion, the eight phenolics with the strongest antioxidant ability reliably represented the bioactivity of the five extracts examined in this study. Moreover, the *Morus alba* hybrid had more phenolic biosynthesis machinery than its cross-parent *M. alba*, whereas the *Broussonetia papyrifera* hybrid had significantly less phenolic machinery than *B. papyrifera*. This difference is probably the main reason for livestock preference for the hybrid of *B. papyrifera* over *B. papyrifera* in feed.

## Introduction

*Morus* and *Broussonetia* tree species (family: Moraceae) have high economic value; among other uses, their leaves are widely used as feed to improve meat quality. The genus *Broussonetia* comprises five species: *B. papyrifera*, *B. kazinoki*, *B. zeylanica*, *B. kaempferi*, and *B. kurzii*^1^, some of which are used as folk medicinal plants and/or for making bark paper^2^. The bark, fruits, leaves, and roots of *B. papyrifera* and *B. kazinoki* exhibit antioxidant, anti-inflammatory, antinociceptive, and anti-tyrosinase activity^3–6^. In China, *B. papyrifera* and *B. kazinoki* leaves, from which several bioactive components such as polyphenols, flavonoids and alkaloids have been isolated^7^, are widely used to feed livestock for meat quality improvement. Methanol extracts from *B. papyrifera* leaves have greater inhibitory effects on mushroom tyrosinase and 2,2-diphenyl-1-picrylhydrazyl/(DPPH)• scavenging activity^8^, and ethanol extracts from *B. papyrifera* roots inhibit both acetyl- and butyl-cholinesterases^2^. Papyriflavonol A, isolated from the root bark of *B. papyrifera*, exhibits strong antimicrobial activity against different pathogenic bacteria and fungi^9^, whereas a 70% ethanol leaf extract of *B. kazinoki* was reported to dramatically reduce ear and dorsal skin thickness and other clinical symptoms in mice with atopic dermatitis, possibly via the downregulation of immunoglobulin E (IgE) and interleukin-4 (IL-4) plasma levels^5^. Broussonone A extracted from *B. kanzinoki* stem bark displays noncompetitive inhibitory activity against pancreatic lipase^10^. However, data on the antioxidant activity and bioactive composition of *B. kanzinoki* remains limited.

The genus *Morus* comprises 10–16 species^11^, of which *M. alba* is the most abundant and economically valuable^12^. *M. alba* is widely used to feed silkworms to produce silk fiber, and to feed animals to produce high-quality meat; extracts from its root bark, leaves, and fruits have been reported to have anti-diabetic, antibacterial, antioxidant, anticancer, anti-inflammatory, cardioprotective, and anti-atherogenic properties^13–16^. Previous studies have discovered various phenolic acids and flavonoids in extracts from *M. alba* leaves, including several caffeoylquinic acids, kaempferol, rutin, and quercetin-3-*O*-glucoside^17^. Phenolic extracts from *M. alba* leaves have been reported to prevent chronic diseases through inhibition of nuclear factor-kB (NF-kB)-mediated inflammatory responses; these pharmaceutical effects may be linked to the phenolic composition of these extracts^18^. For example, rutin has demonstrated cytoprotective effects against H_2_O_2_-induced oxidative cell destruction, as well as antioxidant activity and anti-inflammatory effects^13^. The high economic and medicinal value of *M. alba* and *B. papyrifera* has led to the development of hybrids of *M. alba* and *B. papyrifera*; these hybrids have short growth cycles, strong adaptability, and high protein content, making them suitable for large-scale cultivation and can be used as candidates for developing functional feeds.

Conventional isolation of bioactive compounds is usually performed using high-speed counter-current chromatography and bioassay-guided fractionation^19^. However, these strategies are considered time-consuming, cumbersome, inefficient, and wasteful of organic solvents, while also having low recovery rates and high cost. The antioxidant activity of bioactive compounds is generally evaluated using spectrophotometry methods such as 2,2-diphenyl-1-picrylhydrazyl (DPPH), 2,2′-azino-bis (3-ethylbenzothiazoline-6-sulphonic acid) (ABTS), and ferric reducing antioxidant power (FRAP) assays; however, these approaches are limited to assessing the total antioxidant capacity of the extract and the total antioxidant capacity of the extract is compounded by the antioxidant capacity of individual antioxidants. The ability of each antioxidant to terminate radical chain processes cannot be evaluated directly^20^. Therefore, it is of great importance to develop a rapid and accurate technique to determine the specific ability of individual active compounds in the natural extracts of complexes. High-performance liquid chromatography (HPLC) analysis combined with DPPH and/or ABTS assays are currently implemented for rapid screening of active compounds in extracts^21,22^.

In DPPH/ABTS-guided HPLC analysis, after a specific antioxidant in the extract reacts with DPPH• or ABTS•^+^, one or more hydrogen atoms from the former are transferred to the latter, leading to a decrease in, or extinction of, the antioxidant peak compared to that in the HPLC profile of the unreacted extract^21^. In total, 127 compounds have been identified from the so-called ‘Yangxinshi Tablet’ and 34 antioxidant compounds were rapidly screened using an online DPPH-guided HPLC assay^22^. In another study, an offline DPPH-guided HPLC assay was applied to a Brazilian green propolis extract to screen nine DPPH• scavengers from among 14 characteristic peaks in the HPLC profiles^23^. A combination of DPPH-guided HPLC, diode-array detection, and time of flight mass spectrometry approaches (HPLC–DAD–TOF–MS) was initially used to screen and identify three antioxidants from peanut shells^24^. However, to our knowledge, offline methods has not been used to evaluate the synergistic or antagonistic effects of multiple antioxidants in complex extract mixtures.

The objective of this study was to determine and compare phenolic compositions (i.e., total phenols and flavonoids) in leaf extracts of five tree species in the two genera of the family Moraceae, and to determine their antioxidant abilities via DPPH, ABTS, oxygen radical absorbance capacity (ORAC), and cellular antioxidant activity (CAA) assays. Of the 23 phenolics identified by ultra-performance liquid chromatography quadrupole time of flight tandem mass spectrometry (UPLC–QTOF–MS/MS), 15 were screened by our modified DPPH/ABTS-guided HPLC assays. Structure-activity relationships and possible synergistic effects of the eight strongest phenolic antioxidants were analyzed, and the antioxidant capacity of the five leaf extracts was comprehensively evaluated. The present work is expected to provide references for the chemical composition and biological activity of *M. alba* and *B. papyrifera* hybrids as functional feeds.

## Results and Discussion

### Total phenol and flavonoid characterization and evaluation of antioxidant activity in five extracts

**Figure 1:**
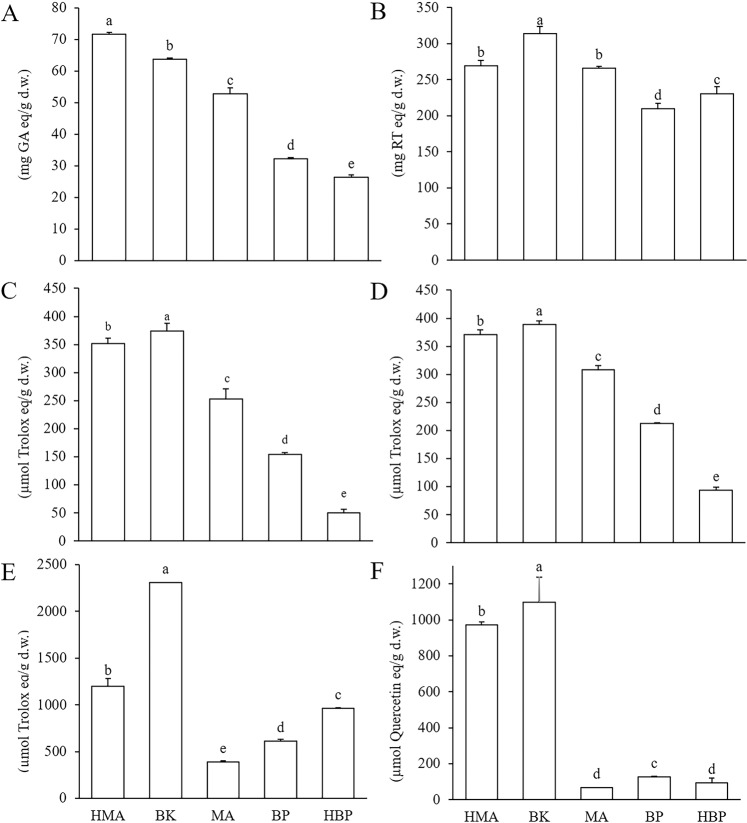
Total phenols, total flavonoids, and antioxidant capacity of leaf extracts from hybrid *Morus alba* L. (HMA), *Broussonetia kazinoki* Sieb (BK), *M. alba* (MA), *B. papyrifera* (L.) Vent. (BP), and hybrid *B. papyrifera* (HBP). Gallic acid was used as the standard for measurement of total phenols (**A**); Rutin for total flavonoids (**B**); Trolox for 2,2-diphenyl-1-picrylhydrazyl (DPPH) (**C**); 2,2′-azino-bis(3-ethylbenzothiazoline-6-sulphonic acid) (ABTS) (**D**); and oxygen radical absorbance capacity (ORAC) assays (**E**); and quercetin for cellular antioxidant activity (CAA) assay (**F**);Results are means ± standard deviation (SD) of three independent experiments (n = 3) and are expressed as mg standard/100 g dry weight (d.w.) (**A**,**B**) or μmol standard/g d.w. (**C**–**F**) Different letters indicate significant differences (*P* < 0.05).

To determine the total phenol content of each of the five extracts, we applied the Folin–Ciocalteu method. Leaves of hybrid *Morus alba* L. (HMA) had the highest total phenol content (71.62 mg gallic acid equivalent [GAE]/g dry weight [d.w.] extract), followed by *Broussonetia kazinoki* Sieb (BK), *M. alba* L. (MA), *B. papyrifera* (L.) Vent. (BP), and hybrid *B. papyrifera* (HBP) at 63.68; 52.71; 3,217.86; and 26.26 mg/GAE/g d.w. extract (Fig. 1A). The highest phenol content in HMA was 2.73-fold that of the lowest phenol content in HBP, and all pairs of adjacent extracts were significantly different (*P* < 0.05). In contrast, there was a 1.50-fold difference between the highest and lowest total flavonoid content, as determined by the aluminum chloride colorimetric assay (Fig. 1B), in the following order (mg rutin/g d.w. extract): BK (313.77) > HMA (269.43) > MA (265.61) > HBP (230.78) > BP (209.62). There was no significant difference in flavonoid content between HMA and MA (*P*** >** 0.05).

We then conducted DPPH, ABTS, ORAC, and CAA assays to evaluate antioxidant activity in the five extracts. DPPH• and ABTS•^+^ scavenging capacities were in the order BK** >** HMA** >** MA** >** BP** >** HBP (Fig. 1C,D), with significant differences between each pair of extracts. The ABTS assay yielded larger values than the DPPH assay, in all extracts except BK. These patterns were comparable to those observed for total phenol content (Fig. 1A), implying that total phenols were the main antioxidants in these two assays. For BK, however, flavonoid content was the main antioxidant in DPPH and ABTS assays. Similar orders of ORAC (BK** >** HMA** >** HBP** >** BP** >** MA; all pairs significantly different, *P* < 0.05) and CAA values (BK** >** HMA** >** BP** >** HBP** >** MA; all pairs significantly different except MA and HBP) were observed (Fig. 1E,F). These similarities suggested that similar mechanisms underlie the antioxidant activity examined by these two assays; the marked differences in ranking order between these assay results and those of the DPPH and ABTS assays may indicate different mechanisms between these groups of assays. Finally, the ranking order for total flavonoid content (Fig. 1B) was similar between the two groups of assays, suggesting that total flavonoids in the five extracts may be the main contributors to antioxidant activity in these two assays.

*Kim et al*.^25^ determined the total phenol content of 10 extracts from *M. alba* leaves and found that the highest content was 55.40 mg GAE/g extract, similar to our result for MA (52.71 mg GAE/g), but much lower than that for HMA (71.62 mg GAE/g). *Mahmoud et al*.^26^ reported total phenol and total flavonoid content in *M. alba* leaves (67.55 mg GAE/g and 39.24 mg rutin/g, respectively), both substantially less than that of HMA in the present study (71.62 mg GAE/g for phenols and 269.43 mg rutin/g for flavonoids) and BK (63.68 for phenols and 313.77 for flavonoids); our results were 6-fold higher. This difference may be attributable to different extraction schemes; we used petroleum ether as a degreasing agent following extraction with 70% ethanol, rather than using only 90% ethanol. *Lee et al*.^5^ investigated total phenol content (22.00 mg GAE/g) and total flavonoid content (24.00 mg rutin/g) in *B. kazinoki* leaf extracts with 70% ethanol; their contents were much lower than ours for BK.

*Wang et al*.^27^ determined antioxidant activity in ethanolic extracts from leaves, stems, and fruits of *M. alba*, and found that leaves had the strongest free radical scavenging ability. *Sánchez-Salcedo et al*.^17^ examined antioxidant capacity in leaves from eight *M. alba* clones using DPPH and ABTS assays. In the current study, both HMA (351.31 for DPPH and 370.55 for ABTS) and BK (37,3.85 for DPPH and 388.71 for ABTS) exhibited stronger antioxidant capacity than the strongest detected among the eight *M. alba* clones. This difference may be attributed to differences in the methods and solvents used during extraction.

*Radojković et al*.^28^ reported ORAC values for *M. nigra* leaves (743.10 µmol Trolox/g dry plant material) that were dramatically lower than those of HMA (1195.39 µmol Trolox/g) and BK (2305.02 µmol Trolox/g) in the present study, because our results are expressed as ratios of extract d.w. rather than of plant material. To our knowledge, this is the first study to determine total phenols and total flavonoids in leaves of HMA and HBP, as well as their antioxidant activity, using DPPH, ABTS, ORAC, and CAA assays, and the first to determine antioxidant activity in BK and BP via ORAC and CAA assays, and in MA via a CAA assay.

### Characterization of extract phenolic composition via UPLC–QTOF–MS/MS

We next performed UPLC–QTOF–MS/MS in both positive and negative ion modes to ensure appropriate ionization of the five extracts. The results showed that negative ion mode (Supplementary Fig. S1A) provided cleaner parent/fragment ion signals, better resolution, and lower background noise than the positive ion mode (Supplementary Fig. S1B). Therefore, leaf extract phenolic compositions in HMA, BK, MA, BP, and HBP were analyzed in negative ion mode, and a total of 23 phenolic compounds (Supplementary Fig. S1 and Table 1) were successfully identified by comparing MS data for an individual peak, including retention time (Rt), experimental *m/z*, molecular formula, error of experimental *m/z*, and MS^2^ fragments, to those of the 15 authentic standards (Supplementary Fig. S2) and/or those retrieved from the MassBank MS database (http://www.massbank.jp) and other available literature, of which 10 were phenolic acids and 13 were flavonoids (Table 1).

**Table 1:** Ultra-performance liquid chromatography–quadrupole time-of-flight tandem mass-spectroscopy (UPLC–QTOF–MS/MS) data of the compounds identified in this study.

No. of compounds	Rt (min)	[M-H]ˉ (m/z)	Error (ppm)	Formula	MS/MS fragments	Proposed compound	Species
**Phenolic acids**							
1	3.106	191.0189	−1.6	C_7_H_11_O_6_	111.0082, 87.0079	Quinic acid ^A^*	b**
2	7.285	315.0714	−0.6	C_16_H_11_O_7_	152.0108, 108.0215	Gentisoyl hexoside ^AB^	abcde
3	9.650	353.0872	−0.3	C_16_H_17_O_9_	191.0550, 179.0338	5-Caffeoylquinic acid^S^***	abcd
4	14.014	337.0909	−4.2	C_16_H_17_O_8_	163.0393	3-*p*-Coumaroylquinic acid^A^	a
5	15.209	353.0876	0.8	C_16_H_17_O_9_	191.0553	3-Caffeoylquinic acid^S^	abc
6	17.050	353.0864	−2.5	C_16_H_17_O_9_	179.0337, 173.0447, 135.0442	4-Caffeoylquinic acid^S^	abcd
7	20.554	337.0908	−4.4	C_16_H_17_O_8_	191.0544, 173.0446, 163.0402	4-*p*-Coumaroylquinic acid^A^	b
8	22.595	337.0922	−0.3	C_16_H_17_O_8_	191.0551, 163.0394	5-*p*-Coumaroylquinic acid^A^	abc
10	26.625	367.1016	−3.5	C_17_H_19_O_9_	193.0499, 173.0439, 93.0321	4-Feruloylquinic acid^A^	bd
22	53.798	515.1180	−1.9	C_25_H_23_O_12_	353.0863, 191.0545, 173.0439	4,5-Dicaffeoylquinic acid^S^	a
**Flavonoids**							
9	23.338	593.1495	−1.9	C_27_H_29_O_15_	473.1076, 353.0637	Vicenin-2^AS^	e
11	29.975	447.0923	−0.9	C_21_H_19_O_11_	357.0603	Isoorientin	b
12	31.461	447.0922	−1.1	C_21_H_19_O_11_	327.0509	Orientin^S^	bd
13	36.806	431.0987	2.1	C_21_H_19_O_10_	311.0549, 283.0612	Vitexin^S^	bde
14	37.699	431.0983	1.2	C_21_H_19_O_10_	341.0671, 311.0563	Isovitexin^S^	bde
15	37.934	609.1453	−0.5	C_27_H_29_O_16_	301.0348	Rutin^S^	ac
16	40.398	463.0894	3.7	C_21_H_19_O_12_	300.0275, 255.0306	Isoquercitrin^S^	ac
17	42.083	461.0719	−0.2	C_21_H_17_O_12_	285.0393	Luteolin-7-*O*-glucuronide^AS^	bde
18	45.503	593.1506	0	C_27_H_29_O_15_	285.0390	Kaempferol-3-*O*-rutinoside^S^	a
19	48.167	447.0926	−0.2	C_21_H_19_O_11_	284.0374	Kaempferol-3-*O*-glucoside^S^	ac
20	49.904	431.0973	−1.2	C_21_H_19_O_10_	269.0421	Apigenin 7-glucoside^S^	bde
21	52.466	445.0775	0.9	C_21_H_17_O_11_	269.0450	Apigenin-7-*O*-glucuronide^AS^	bde
23	55.805	489.1030	−0.6	C_23_H_21_O_12_	285.0388, 191.0567	Kaempferol-3-*O*-6″-*O*-acetyl-β-D-glucopyranoside^B^	a

*A and B are reported for the first time in the genera *Broussonetia* and *Morus*, respectively.

**a, b, c, d, and e were identified from leaf extracts of the hybrid *Morus alba*, *Broussonetia kazinoki*, *M. alba*, *B. papyrifera*, and the *B. papyrifera* hybrid, respectively.

***S is identified by standard.

### Identification of the 10 phenolic acids

UPLC–QTOF–MS/MS data of the compounds detected in this study are shown in Table 1. According to *Fu et al*.^29^, compound 2 was tentatively assigned as gentisoyl hexoside; to the best of our knowledge, this is the first report of gentisoyl hexoside detection in *Broussonetia* species and HMA. Following comparison of ultraviolet (UV) and MS data, we unambiguously identified compound 1 as quinic acid, which is an essential component of various feruloyl-, coumaroyl-, and caffeoylquinic acid derivatives^30^. According to a previous report^31^, the base peak of 3-feruloylquinic acid and 5-feruloylquinic acid were 191, while the base peak of compound 10 was 173, and we identified compound 10 as 4-feruloylquinic acid. To our knowledge, this is the first study to detect 4-feruloylquinic acid in *Broussonetia*. MS data for compounds 4, 7, and 8 indicated the presence of *p*-coumaroylquinic acid isomers; following a comparison of column retention behavior and other data from previous reports^31,32^, we identified these compounds as 3-, 4-, and 5-*p*-coumaroylquinic acid, respectively. These three *p*-coumaroylquinic acids in BK Mass data for compounds 3, 5, and 6 indicated the presence of monocaffeoylquinic acid isomers; following comparison of column retention behavior and MS data between the authentic standards and those previously reported^33^, these compounds were identified as 5-, 3-, and 4-caffeoylquinic acid, respectively (Table 1). These monocaffeoylquinic acids were previously identified in *M. alba* leaves^11,17^, but not in BK leaves; 5- and 4-caffeoylquinic acids were previously reported in BP leaves. Compound 22 was characterized as 4,5-dicaffeoylquinic acid because it showed an [M-H]^−^ ion at *m/z* 515 and a fragment anion [M-H-162]^−^ at *m/z* 353, corresponding to the loss of a caffeoyl (Table 1); this compound was previously identified in *M. alba* leaves^11^.

### Identification of the 13 flavonoids

As shown in Table 1, eight flavonoids (compounds 9, 11, 12, 13, 14, 17, 20, and 21) were identified from *Broussonetia*; the other five flavonoids (compounds 15, 16, 18, 19, and 23) were identified from *Morus*. Interestingly, these two genera belonging to the same family contained flavonoids with completely different structures.

Based on reports by *Dugo et al*.^11^ and *Benayad et al*.^34^, and the MS data for their standards, compounds 9 and 15–17 herein were determined as vicenin-2, rutin, isoquercitrin, and luteolin-7-*O*-glucuronide, respectively. Compound 9 has not previously been reported in HBP; in the present study, it was identified only in HBP. Compound 17 was detected for the first time in BK, BP, and HBP leaves.

Following comparison of typical MS^2^ fragments in the standards with those previously reported^7,35^, compounds 11–14 and 18–21 were identified as isoorientin, orientin, vitexin, isovitexin (vitexin and isovitexin are a pair of isomers), kaempferol-3-*O*-rutinoside, kaempferol-3-*O*-glucoside, apigenin 7-glucoside, and apigenin-7-*O*-glucuronide, respectively. Compounds 12–14 have been reported in BP leaves^7,35^; however, to our knowledge, this is the first study to report compounds 11–14 in BK and compounds 13 and 14 in HBP. Compounds 18 and 19 have been identified in *M. alba* leaves but are reported in HMA for the first time^11^. Compound 20 has been reported in BP leaves^35^, but not in BK and HBP, and compound 21 is reported in both for the first time.

Finally, compound 23 showed an [M-H]^−^ ion at *m/z* 489; its MS^2^ yielded a fragment at *m/z* 285 due to the loss of a sugar and an acetyl (Table 1). Following comparison with data from the MassBank MS database, we tentatively identified it as kaempferol-3-*O*-6″-*O*-acetyl-β-D-glucopyranoside, which has not been identified previously in *M. alba* leaves.

### Screening of active antioxidants by DPPH/ABTS-guided HPLC analysis

The above results indicate strong antioxidant activity among extracts from the five Moraceae species or hybrids, and 23 phenolic substances were identified via UPLC–QTOF–MS/MS. We next used DPPH/ABTS-guided HPLC assays, a rapid method for screening active antioxidant compounds from complex mixture extracts^36^, to screen effective antioxidants and evaluate the contribution of individual compounds to total antioxidant activity within a complex mixture extract^21^. When an antioxidant compound reacts with a radical such as DPPH• or ABTS•^+^, the result is a redox reaction; i.e., the molecular structure of the antioxidant changes^37^, with the peak area (PA) becoming significantly smaller in the chromatogram^38^. Among the antioxidant compounds in the mixture extract, a greater change in PA due to reaction with the free radical indicates a greater contribution of the compound to the antioxidant activity of the extract.

**Figure 2:**
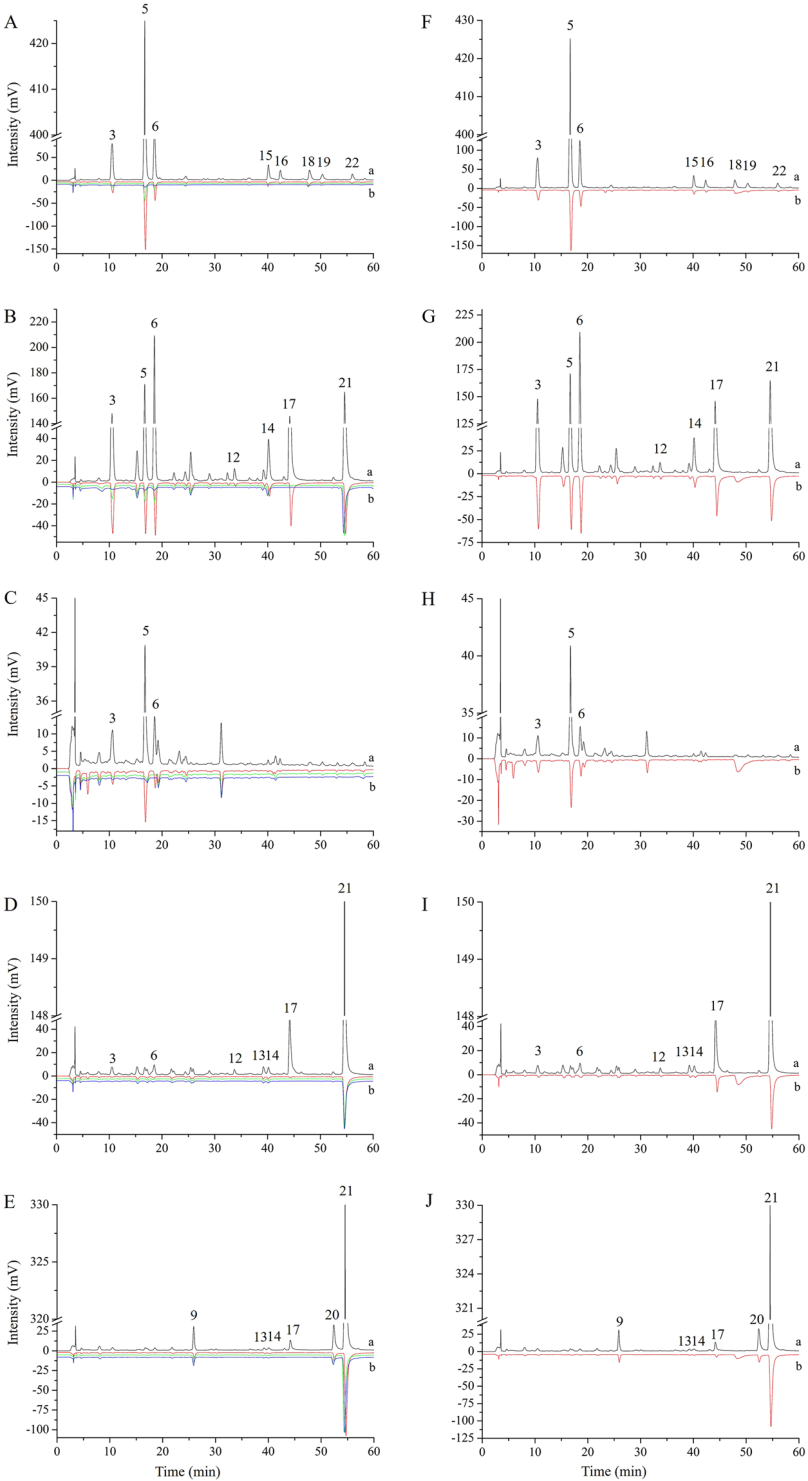
High-performance liquid chromatography (HPLC) chromatograms of hybrid *Morus alba* L. (HMA), *Broussonetia kazinoki* Sieb (BK), *M. alba* (MA), *B. papyrifera* (L.) Vent. (BP), and hybrid *B. papyrifera* (HBP) before (a) and after (b) reaction with DPPH• (**A**–**E**) and ABTS•^+^ (**F**–**J**). Red, green, and purple curves in (**A**–**E**) represent DPPH• concentrations of 1, 5, and 10 mM, respectively. Red curves in (**F**–**J**) represent 7 mM ABTS•^+^.

We screened 15 antioxidant compounds identified by UPLC–QTOF–MS/MS using DPPH/ABTS-guided HPLC assays: compounds 3 (5-caffeoylquinic acid), 5 (3-caffeoylquinic acid), 6 (4-caffeoylquinic acid), 9 (vicenin-2), 12 (orientin), 13 (vitexin), 14 (isovitexin), 15 (rutin), 16 (isoquercitrin), 17 (luteolin-7-*O*-glucuronide), 18 (kaempferol-3-*O*-rutinoside), 19 (kaempferol-3-*O*-glucoside), 20 (apigenin 7-glucoside), 21 (apigenin-7-*O*-glucuronide), and 22 (4,5-dicaffeoylquinic acid) (Fig. 2; Table 2). Based on the extent of reduction in PA following reaction with DPPH• or ABTS•^+^, the 15 phenolic compounds were divided into two categories: 8 strong antioxidants (compounds 3, 5, 6, 12, 16, 15, 17, and 22) and 7 weak antioxidants (9, 13, 14, 18, 19, 20, and 21).

**Table 2:** Free radical scavenging activity of the 15 phenolic compounds of the five extracts examined in this study.

Species	Peak no.	Phenolic compound	DPPH			ABTS
			PK1	PK5	PK10	PK7
MA	3	5-Caffeoylquinic acid	67.95 ± 2.59^b^	84.31 ± 0.86^a^	90.97 ± 2.17^a^	53.70 ± 2.03
	5	3-Caffeoylquinic acid	66.87 ± 2.72^b^	91.58 ± 2.07^a^	94.69 ± 1.34^a^	49.70 ± 1.91
	6	4-Caffeoylquinic acid	72.01 ± 3.06^b^	79.24 ± 3.61^ab^	83.29 ± 1.85^a^	54.92 ± 2.37
	15	Rutin	84.08 ± 1.17^c^	87.36 ± 0.59^b^	90.09 ± 0.46^a^	75.38 ± 0.94
	16	Isoquercitrin	87.89 ± 1.81^b^	100^a^	100^a^	80.84 ± 1.21
	19	Kaempferol-3-*O*-glucoside	89.29 ± 1.36^c^	70.38 ± 1.37^b^	93.84 ± 1.02^a^	–*
HMA	3	5-Caffeoylquinic acid	70.15 ± 0.68^c^	86.94 ± 1.21^b^	96.74 ± 0.73^a^	67.87 ± 0.44
	5	3-Caffeoylquinic acid	65.23 ± 0.83^c^	90.08 ± 0.75^b^	97.02 ± 0.92^a^	63.18 ± 0.64
	6	4-Caffeoylquinic acid	68.56 ± 1.29^c^	89.23 ± 0.82^b^	97.32 ± 1.27^a^	67.09 ± 1.37
	15	Rutin	69.41 ± 5.16^b^	98.66 ± 0.06^a^	98.77 ± 0.06^a^	64.02 ± 1.10
	16	Isoquercitrin	79.28 ± 3.97^b^	98.92 ± 0.32^a^	100^a^	77.06 ± 4.13
	18	Kaempferol-3-*O*-rutinoside	57.50 ± 1.80^c^	75.87 ± 0.31^b^	80.98 ± 0.85^a^	14.97 ± 8.84
	19	Kaempferol-3-*O*-glucoside	73.78 ± 2.47^b^	88.62 ± 0.28^a^	92.48 ± 0.39^a^	46.91 ± 1.52
	22	4,5-Dicaffeoylquinic acid	74.65 ± 0.49^b^	97.18 ± 1.52^a^	97.91 ± 0.30^a^	70.11 ± 0.62
BP	3	5-Caffeoylquinic acid	87.59 ± 4.45^a^	94.72 ± 0.28^a^	93.84 ± 1.75^a^	75.92 ± 3.86
	6	4-Caffeoylquinic acid	83.80 ± 3.35^b^	92.98 ± 0.30^a^	92.98 ± 0.69^a^	78.47 ± 5.08
	12	Orientin	98.90 ± 0.29^b^	100^a^	100^a^	82.94 ± 0.85
	13	Vitexin	76.34 ± 0.88^a^	77.27 ± 0.64^a^	77.37 ± 1.95^a^	75.31 ± 0.58
	14	Isovitexin	75.08 ± 0.91^a^	77.65 ± 0.12^a^	77.82 ± 3.17^a^	74.70 ± 2.49
	17	Luteolin-7-*O*-glucuronide	98.43 ± 0.54^b^	100^a^	100^a^	78.44 ± 0.86
	21	Apigenin-7-*O*-glucronide	69.70 ± 0.15^a^	70.93 ± 0.45^a^	71.46 ± 0.85^a^	69.45 ± 0.30
HBP	9	Vicenin-2	62.50 ± 0.75^b^	63.95 ± 0.60^ab^	65.20 ± 0.44^a^	62.15 ± 1.58
	13	Vitexin	70.14 ± 0.58^a^	69.45 ± 1.37^a^	70.74 ± 1.07^a^	67.78 ± 3.08
	14	Isovitexin	67.82 ± 1.84^a^	69.63 ± 1.35^a^	71.63 ± 1.46^a^	67.63 ± 2.55
	17	Luteolin-7-*O*-glucuronide	100^a^	100^a^	100^a^	76.30 ± 0.93
	20	Apigenin 7-glucoside	67.47 ± 0.56^b^	68.07 ± 0.93^b^	70.24 ± 1.07^a^	63.86 ± 2.26
	21	Apigenin-7-*O*-glucuronide	63.73 ± 0.74^a^	65.52 ± 0.75^a^	68.13 ± 2.85^a^	63.28 ± 1.03
BK	3	5-Caffeoylquinic acid	67.03 ± 0.92^c^	89.30 ± 1.23^b^	96.78 ± 0.82^a^	59.66 ± 0.74
	5	3-Caffeoylquinic acid	72.40 ± 0.20^c^	90.01 ± 0.69^b^	96.25 ± 0.73^a^	65.18 ± 0.21
	6	4-Caffeoylquinic acid	76.49 ± 1.44^c^	91.01 ± 0.69^b^	96.85 ± 0.66^a^	69.75 ± 1.08
	12	Orientin	73.11 ± 2.61^b^	99.35 ± 0.18^a^	100^a^	67.82 ± 1.06
	14	Isovitexin	67.88 ± 1.15^c^	72.84 ± 1.85^b^	77.38 ± 1.03^a^	66.26 ± 0.71
	17	Luteolin-7-*O*-glucuronide	72.17 ± 0.87^b^	99.29 ± 0.24^a^	99.94 ± 0.01^a^	68.88 ± 0.57
	21	Apigenin-7-*O*-glucuronide	69.12 ± 0.18^a^	72.37 ± 8.43^a^	67.76 ± 1.49^a^	67.22 ± 1.40

PK1, PK5, PK10, and PK7: free radical scavenging rates (%) of individual phenolic compounds after reaction with 1, 5, and 10 mM 2,2-diphenyl-1-picrylhydrazyl (DPPH) and 7 mM 2,2′-azino-bis (3-ethylbenzothiazoline-6-sulphonic acid) (ABTS), respectively. Results are means ± SD of three independent experiments (n = 3). Different letters indicate significant differences between PK1, PK5 and PK10 (*P* < 0.01). –*: signal covered by the ABTS peak.

### DPPH-guided HPLC analysis

To visually evaluate the free radical scavenging ability of an antioxidant, we modified the DPPH-guided HPLC assay on the basis of a previous study^39^; we used three different concentrations of DPPH•, and the free radical scavenging ability of each antioxidant was determined based on the extent to which its PA decreased as the DPPH• concentration increased. Figure 2A–E shows the HPLC profiles of HMA, BK, MA, BP, and HBP leaf extracts before (upright) and after (reversed) reaction with 1.0, 5.0, and 10 mM DPPH•. FRSRs of individual antioxidants in an extract after reaction with DPPH• (Table 2) were calculated as described in materials and methods.

For the three tested DPPH• levels, FRSRs of compounds 3, 5, 6, and 18 from HMA, and compounds 3, 5, 6, and 14 from BK, were significantly enhanced in a dose-dependent manner; however, no significant differences were observed in the FRSRs of compound 21 from BK, compounds 3, 13, 14, and 21 from BP, and compounds 13, 14, 17, and 21 from HBP, among which compound 21 from BK and compounds 17 and 21 from HBP had the highest FRSRs (69, 100, and ~65%, respectively) at the lowest DPPH• level of 1 mM (Table 2). FRSRs of compounds 15, 16, 19, and 22 from HMA, compounds 12 and 17 from BK, compounds 3 and 5 from MA, and compounds 6, 12, and 17 from BP had significantly enhanced FRSRs at only the two lowest DPPH• levels, among which FRSRs of compound 16 from HMA, compounds 12 and 17 from BK, and compounds 12 and 17 from BP approached or attained 100% at the medium DPPH• level of 5 mM. Finally, compound 6 from MA and compound 9 from HBP showed significantly different FRSRs only at DPPH• levels between 1 and 10 mM, and compound 20 from HBP did so only between 5 and 10 mM. The compounds were ordered as follows in terms of FRSRs (highest to lowest): 17, 12, 16, 15, 22, 3, 5, 6, 19, 14, 13, 18, 21, 20, and 9.

Several compounds identified from multiple extracts showed identical FRSRs including compounds 3, 5, and 6 from HMA, BK, MA, and BP (compound 5 was not detected from BP), compounds 14, 17, and 21 from BK, BP, and HBP, and compound 13 from BP and HBP, confirming that antioxidant activity was relatively stable in these compounds in mixed extracts from the five different tree species. In contrast, compounds 12 and 17 from BK and BP showed identical FRSRs at 5 and 10 mM (99–100%), but significantly different FRSRs at the lowest DPPH• level of 1 mM (72–73% in BK and 98–99% in BP), indicating a synergistic effect among phenolics.

### ABTS-guided HPLC analysis

The ABTS-guided HPLC assay is commonly used to screen bioactive antioxidants from complex mixed extracts. Chromatograms of the five extracts before and after reaction with 7 mM ABTS•^**+**^ are shown in Fig. 2F–J; most peaks significantly decreased in size, or disappeared, after the reaction. As shown in Table 2, FRSRs for spiking ABTS•^**+**^ were much lower than those for DPPH• scavenging, and clearly reflected differences in analytes among the five extracts. The compounds were ordered as follows in terms of FRSRs (highest to lowest): 12, 6, 17, 16, 3, 13, 14, 22, 5, 21, 15, 20, 9, 19, and 18; this pattern was similar to that observed for the DPPH• peaks.

Compounds identified from multiple extracts exhibited different ABTS•^**+**^ scavenging abilities: FRSRs of compounds 3, 5, and 6 from HMA and BK were similar, and were much higher and lower than those from MA and BP, respectively; compound 5 was not detected from BP. Compounds 14, 17, and 21 were identified from BK, BP, and HBP, with significantly higher FRSRs in BP than in the other two extracts. Finally, the FRSRs of compounds 12 and 13 from BP were much higher than those from BK and HBP, respectively. Together, these differences indicate synergistic effects among phenolic compounds.

### Antioxidant ability and structure-activity relationships among the 15 phenolics

#### DPPH• and ABTS•^+^ antioxidant ability

**Figure 3:**
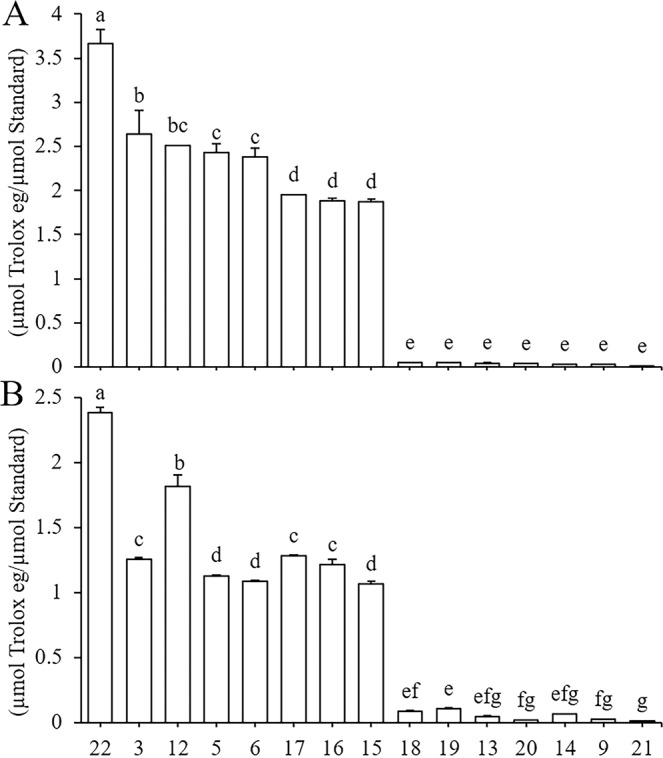
Antioxidant capacity of 15 authentic standards determined by DPPH (**A**) and ABTS (**B**) assays. (22: 4,5-dicaffeoylquinic acid, 3: 5-caffeoylquinic acid, 12: Orientin, 5: 3-caffeoylquinic acid, 6: 4-caffeoylquinic acid, 17: Luteolin-7-*O*-glucuronide, 16: Isoquercitrin, 15: Rutin, 18: Kaempferol-3-*O*-rutinoside, 19: Kaempferol-3-*O*-glucoside, 13: Vitexin, 20: Apigenin-7-glucoside, 14: Isovitexin, 9: vicenin-2, 21: Apigenin-7-*O*-glucuronide). Results are means ± SD of three independent experiments (n = 3) and are expressed as μmol Trolox eq/μmol standard phenolic compound. Different letters indicate significant differences (*P* < 0.01). The order of phenolics from left to right along the x-axis corresponds to DPPH values from highest to lowest.

To evaluate the reliability of the antioxidant activity results for each phenolic compound in the extract mixture obtained using DPPH/ABTS-guided HPLC assays, we determined the antioxidant efficacy of each compound via traditional DPPH and ABTS spectrophotometric assays (Fig. 3).

As shown in Fig. 3A, compound 22 (3.664 µmol Trolox/µmol standard) had the strongest DPPH• scavenging ability, which was significantly higher than that of compound 3 (2.644), followed by compounds 12 (2.509), 5 (2.431), 6 (2.378), 17 (1.948), 16 (1.880), and 15 (1.873). There were no significant differences among compounds 3 and 12; 12, 5, and 6; or 17, 16, and 15 (*P* > 0.01). The remaining seven compounds were all flavonoids having a single hydroxyl on the B-ring: compounds 18 (0.048), 19 (0.047), 13 (0.043), 20 (0.038), 14 (0.032), 9 (0.025), and 21 (0.005). No significant differences were detected among these compounds (*P* > 0.01).

According to the results of the ABTS assay, the scavenging activity pattern of these 15 antioxidants (Fig. 3B) was roughly similar to that determined by the DPPH method (Fig. 3A). Interestingly, the order of the 11 flavonoids in terms of antioxidant activity was exactly the same with both assays. The highest ABTS value was exhibited by compound 22 (2.386), followed by compounds 12 (1.818), 17 (1.281), 3 (1.256), 16 (1.218), 5 (1.131), 6 (1.086), and 15 (1.068). No significant differences were observed among 3, 17, and 16, or among 5, 6, and 15 (*P* > 0.01). The remaining compounds were ranked as follows: compounds 19 (0.108) > 18 (0.085) > 14 (0.065) > 13 (0.046) > 9 (0.023) > 20 (0.020) > 21 (0.013). There were no significant differences among these seven compounds (*P* > 0.01), with the exception of compound 19.

Overall, among the 15 phenolic compounds, 8 had clearly higher free radical scavenging ability than the remaining 7 (Fig. 3). The eight phenolic compounds with the strongest scavenging ability included all four caffeoylquinic acids: compounds 22, 6, 5, and 3. All flavonoids among these compounds had double hydroxyls on the B-ring: compounds 12, 17, 16, and 15. The remaining seven phenolic compounds with weak scavenging ability were flavonoids with a single hydroxyl on the B-ring; there were almost no significant differences in free radical scavenging ability among these compounds (*P* > 0.01).

#### Structure-activity relationships

The above results (Fig. 3) suggest that the position at which caffeoyl groups attach to quinic acid, and the presence of two hydroxyl groups instead of one on the flavonoid B-ring, greatly enhanced scavenging ability in both DPPH• and ABTS•^+^. In contrast, the attachment of glucosides and glucuronides to flavonoids decreased free radical scavenging ability to some extent.

**Figure 4:**
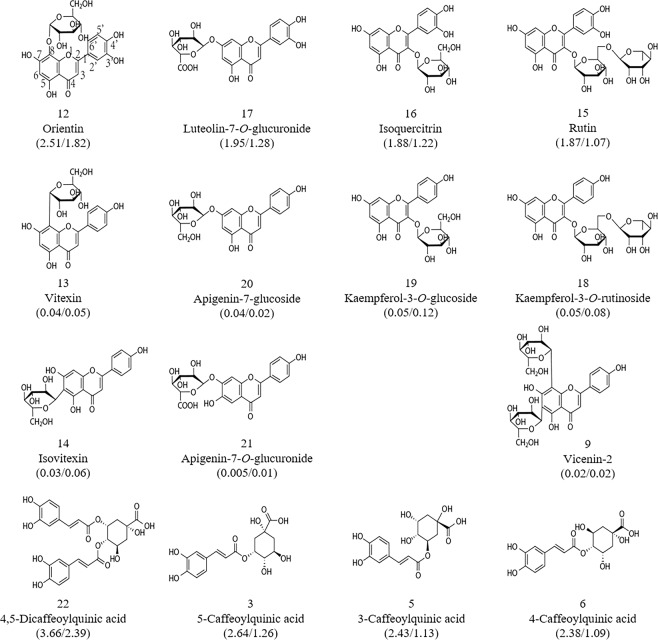
Chemical structures of the 15 phenolics with the highest free radical scavenging rates (FRSRs), as evaluated by DPPH/ABTS-guided HPLC assays. The four flavonoids in the first row have double hydroxyl groups on the B-ring and showed stronger antioxidant activity; differences in antioxidant activity among orientin, isoquercitrin, and rutin were caused by the position and number of sugar groups. The four flavonoids in the second row and three flavonoids in the third row have a single hydroxyl group on the B-ring and showed the weakest antioxidant activity; differences in antioxidant activity among vitexin, kaempferol-3-*O*-glucoside, kaempferol-3-*O*-rutinoside, isovitexin, and vicenin-2 were caused by the position and number of glycosyl groups on the A- and C-rings. The last row contains four caffeoylquinic acids; the first of these is dicaffeoylquinic acid, which showed the strongest antioxidant activity, followed by three monocaffeoylquinic acids, in which antioxidant activity was affected by the position of the caffeoyl group on the quinic acid. The number above the compound name represents the compound number consistenting with that in Table 1, and numbers in parentheses under each compound represents DPPH/ABTS antioxidant capacity.

*Senthil Kumar* and *Kumaresan et al*.^40^ reported that the presence of an ortho-(3′,4′-) dihydroxy structure on the flavonoid B-ring strongly influences free radical scavenging ability, probably because the B-ring acts as a hydrogen atom donor and the relatively stable flavonoid radical is formed through electron delocalization^41^. As shown in Fig. 3, all four flavonoids with an ortho-dihydroxy structure on the B-ring showed significantly higher antioxidant activity than the remaining seven flavonoids, which did not possess this dihydroxy structure. The most prominent differences in antioxidant activity were observed, respectively, between compounds 12, 17, 16, and 15, which have the ortho-dihydroxy structure on the B-ring, and compounds 13, 20, 19, and 18, which have a single hydroxyl group on the B-ring (Fig. 4). These four pairs of flavonoids have the same structure except for this single difference in the B-ring; however, the resulting difference in antioxidant activity was at least 10-fold (Fig. 4), providing strong and direct evidence that the ortho-dihydroxy structure on the B-ring contributes greatly to flavonoid antioxidant capacity.

Previous studies have shown that glycosylation of the hydroxyl groups in flavonoids weakens their antioxidant activity, possibly due to the destruction of ortho-hydroxyl structures and/or a reduction in the number of hydroxyl groups^42,43^. The attachment of sugars may prevent the entry of free radical scavengers into the radical center of ABTS•^+^ and DPPH•; thus, sugar moieties may negatively affect antioxidant capacity in various flavonoids^44^. Our comparison of antioxidant ability and structure between compounds 12 and 16 (Figs. 3 and 4) indicated that the sugar moiety negatively affected flavonoid antioxidant capacity to a greater extent on the C-ring than on the A-ring. Our comparison between compounds 16 and 15 (Figs. 3 and 4) clearly showed that the presence of two sugar moieties attached to the C-ring had a greater negative impact than a single sugar moiety. Similarly, comparing compounds 20 and 21 (Figs. 3 and 4) revealed that compound 21 possesses one more hydroxyl group on the A-ring, which had a positive effect on its antioxidant ability, which was similar to that of compound 20. Thus, the glucuronide moiety attached to 7-C on the A-ring weakened the antioxidant ability to a much greater extent than the attachment of a glucoside moiety at exactly the same position. To our knowledge, this represents the first evidence of the effects of glucuronide and glucoside moieties on antioxidant activity. We therefore hypothesize that the glucuronide moiety has a greater effect than the glucoside group in reducing flavonoid antioxidant activity.

Our results revealed higher antioxidant activity in compound 22 with two caffeic acid groups than in the other three monocaffeoylquinic acids (compounds 6, 3, and 5; Figs. 3 and 4); the antioxidant activity of caffeoyl quinic acid is likely attributable to the presence of more caffeoyl groups^45^.

### Synergistic effects and antioxidant activity of the five extracts

#### Synergistic effects of eight high-antioxidant activity phenolics

**Figure 5:**
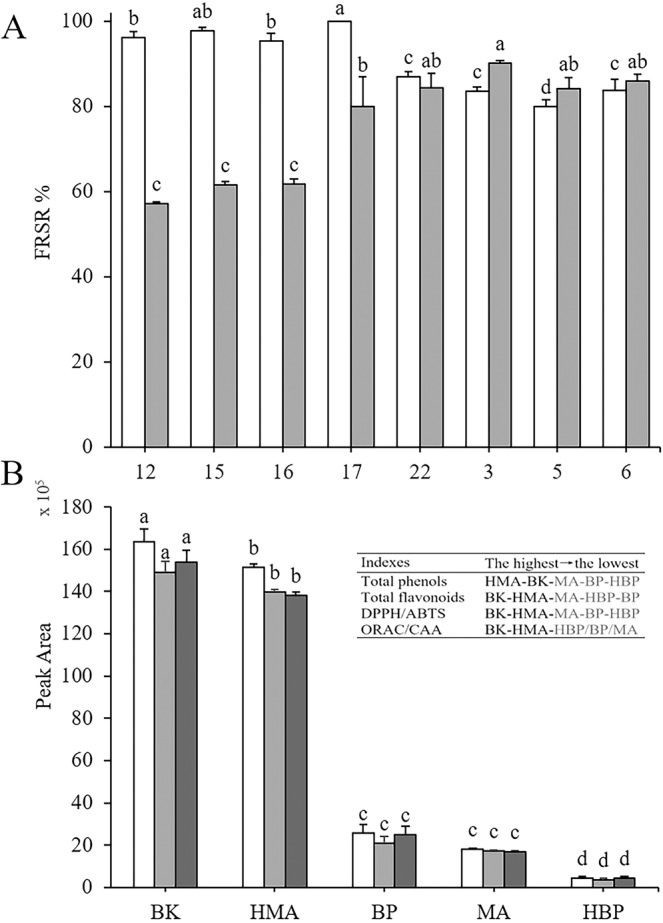
FRSRs of eight authentic standard phenolics determined by DPPH-guided HPLC assay (**A**) and free radical scavenging abilities of the eight strongest antioxidants among the five extracts (**B**). (**A**) The left column shows the FRSRs of a single phenolic compound evaluated by DPPH-guided HPLC assay after reaction with 5 mM DPPH• and the right column shows those from a mixture of the eight phenolics. (12: Orientin, 15: Rutin, 16: Isoquercitrin, 17: Luteolin-7-*O-*glucuronide, 22: 4,5-dicaffeoylquinic acid, 3: 5-caffeoylquinic acid, 5: 3-caffeoylquinic acid, 6: 4-caffeoylquinic acid). (**B**) For each extract, the left column represents DPPH free radical scavenging ability, evaluated as the sum of the product of each PA in the eight phenolics occurring in the extract without reacting with DPPH• × the corresponding FRSRs when the extract reacted with 5 mM DPPH; middle column, × the corresponding FRSRs in the mixture of eight phenolic standards; and right column, × the corresponding FRSRs of the eight phenolic standards. The higher the peak area, the stronger the free radical scavenging ability of the extract. The data in the table at the top right indicate the order of the total phenols, total flavonoids, DPPH/ABTS and ORAC/CAA in the 5 extracts determined by chemical experiments from high to low. BK (*Broussonetia kazinoki* Sieb), HMA (hybrid *Morus alba* L.), BP (*B. papyrifera* (L.) Vent.), MA (*M. alba* L.), and HBP (hybrid *B. papyrifera*). Data shown in (**B**) were recalculated from Supplementary Table S1, Table 2, and Fig. 5A. Results are means ± SD of three independent experiments (n = 3) and are expressed as percentages. Different letters indicate significant differences (*P* < 0.01).

To verify whether synergistic effects are present in the eight phenolics exhibiting the strongest antioxidant activity, we performed a DPPH-guided HPLC assay to evaluate the free radical scavenging ability of these compounds. As shown in Fig. 5A, when each compound reacted with DPPH•, the four flavonoids showed significantly higher FRSRs than the four caffeoylquinic acids, with no significant differences among three of the four flavonoids (*P*** >** 0.01), except compound 17, which showed significant differences with compounds 12 and 16; moreover, there were no significant differences among three of the four caffeoylquinic acids, except compound 5, which exhibited significant differences to the other three. However, in a mixture of the compounds, FRSRs of the four flavonoids were significantly lower than those of the four phenolic acids, and there were no significant differences (*P*** >** 0.01) among three of the four flavonoids, except compound 17, or among the four caffeoylquinic acids. The FRSRs of the four flavonoids in the compound mixture were approximately 40% lower (with the exception of ~20% for compound 17) than those of the individual compounds. However, the FRSRs of the four individual caffeoylquinic acids were similar to those measured in the compound mixture. Therefore, we suspect that other compounds in the mixture may have exerted a negative synergistic or antagonistic effect on the antioxidant ability of the four flavonoids, but exerted no effect on the caffeoylquinic acids.

#### Comprehensive evaluation of the antioxidant capacity of the five extracts

Figure 5B shows the comprehensive free radical scavenging ability of the five extracts, in terms of the eight strongest phenolics in each extract. On average, free radical scavenging ability was 8-fold higher in BK and HMA than in BP, MA, and HBP; the overall ranking in terms of free radical scavenging ability was generally consistent with that of total phenols, total flavonoids, and antioxidant activity among the five extracts (see table in Fig. 5B), suggesting that the DPPH/ABTS-guided HPLC assays reliably screened the eight strongest bioactive phenolics. These results also indicate that hybridization allowed HMA to gain considerably more phenolic biosynthesis machinery compared to its cross-parent MA, whereas HBP lost a significant amount of phenolic biosynthesis machinery compared to BP. This process is likely the main reason for the greater palatability of HBP compared to BP, and explains the preference for leaves of this species among pigs, cattle, and sheep.

## Methods

### Chemicals and leaf extract preparation

Folin–Ciocalteu reagent, Trolox, DPPH, ABTS, ultra-pure water, acetonitrile, formic acid for HPLC, and other chemicals were obtained following *Yang et al*.^20^ We purchased 15 authentic standards from Chengdu Pfeide Biotechnology Co., Ltd. (Chengdu, China).

On May 20, 2018, we collected leaves from *B. papyrifera* (L.) Vent. (BP), *B. kazinoki* Sieb (BK), *M. alba* L. (MA), and commercialized hybrids of *B. papyrifera* (HBP) and *M. alba* (HMA) from Haidian District, Beijing, China. Leaves were washed with purified water and placed in a ventilation oven at 105 °C for 15 min for de-enzyming, and then dried at 60 °C for 24 h in the same oven. Dried leaves were ground, and the powder was sifted through a 1-mm mesh and stored at −20 °C for further extraction. To prepare each extract, 150 mL 70% ethanol was added to 10 g dry leaf powder, and the mixture was sonicated for 30 min at room temperature (power, 500 W; frequency, 40 kHz; KQ-300DE NC ultrasonic cleaner; Kunshan Ultrasonic Instrument Co., Ltd., Jiangsu, China). The sonicated mixture was then filtered using a 0.22-µm filter, and the supernatant was collected. This extraction procedure was repeated twice; the three resulting supernatants were combined and evaporated by rotation at 45 °C until one third of the volume remained. The remaining solution was degreased with petroleum ether^35^, and the lower layer was collected. The collected solutions were dried in a ventilation oven at 60 °C, and the five extracts were collected and stored at −20 °C for further experiments.

### Determination of total phenols, total flavonoids, and antioxidant activity

Total phenols and total flavonoids in the five extracts were measured following the Folin–Ciocalteu and aluminum chloride colorimetric methods^20^. DPPH• and ABTS•^**+**^ scavenging activities were assessed using our modified method^46^. An ORAC assay was performed by modifying a method described previously^47^. A CAA assay was performed as described previously^20^, with minor modifications. All assays were performed in triplicate.

### HPLC and UPLC–QTOF–MS/MS analyses of phenolic composition

HPLC analyses were performed using a Shimadzu (Kyoto, Japan) HPLC system as described previously with slight modifications^17,48^. Briefly, an extract or the mixture of 15 standards was dissolved in 70% ethanol at 0.005 and 0.5 mg/mL, respectively, and then filtered through a 0.22-µm filter before injection. A reversed-phase column (Diamonsil C_18_ 5 µm 250 × 4.6 mm i.d.; Dikma, Beijing, China) was used for separation; the column temperature was set at 30 °C. Two solvents were applied for elution: water containing 0.4% (v/v) formic acid (A) and acetonitrile (B). The gradient started with 8% solvent B, reaching 15% at 25 min, 22% at 55 min, and 40% at 60 min. The flow rate was set at 1.0 mL/min, and the injection volume was 20 µL. The detection wavelength was set at 320 nm to monitor all polyphenols simultaneously. Before each run, the column was equilibrated for 15 min under the initial conditions.

The UPLC–QTOF–MS/MS system comprised an Acquity UPLC system (Waters, Milford, MA, USA) and a QTOF–MS mass spectrometer (Xevo G2-XS; Waters). The C_18_ used for separation was described above, with a column temperature of 30 °C and a mass range of 100–1,000 *m/z*. MS experiments were performed in both positive and negative ionization modes under the following conditions: nitrogen drying gas flow, 10.0 L/min; nebulizer pressure, 45 psi; gas drying temperature, 370 °C; capillary and fragment voltage, 2.500 kV; and MS/MS collision energy, 20 V.

### DPPH/ABTS-guided HPLC assays for active antioxidant screening

DPPH/ABTS-guided HPLC assays were performed as described previously with slight modifications^24,36,39^. Briefly, each of the five extracts was mixed with a different concentration of DPPH• (1, 5, or 10 mM) or ABTS•^+^ (7 mM) solution at a ratio of 1:1 (v:v) to react at room temperature in the dark for 30 min. The extract solution (5 mg/mL) without DPPH• or ABTS•^+^ was used as a control; Vc was used as a radical scavenger reference compound^49^. All evaluated solutions were filtered through a 0.22-μm microporous organic membrane filter prior to HPLC analysis.

The scavenging capacity of DPPH• and ABTS•^+^ for each compound in each mixture extract was expressed as the free radical scavenging rate (FRSR), calculated as follows: FRSR (%) = (*PA*_0_ – *PA*_1_)/*PA*_0_ × 100 (Equation 1), where *PA*_0_ is the area of an individual peak in the profile prior to reaction of the extract with DPPH• or ABTS•^+^, and *PA*_1_ is the area after the reaction.

The peak area (PA), representing the contribution rate of each of the eight strongest phenolics to an extract, was calculated as follows: PA = *PA*_0_ × *FRSR*, where *PA*_0_ (Equation 2) is the area of an individual peak in the profile of the extract prior to reaction with DPPH•.

### Statistical analyses

All experimental results are expressed as means ± standard deviation (SD), and data were analyzed by one-way analysis of variance (*P* < 0.05 or 0.01) using SPSS software (ver. 17.0; SPSS Inc., Chicago, IL, USA).

## Conclusion

We investigated the phenolic composition and antioxidant activities of *B. papyrifera*, *B. kazinoki*, *M. alba*, and commercialized hybrid of *B. papyrifera* and that of *M. alba*. Among the five leaves extracts, the hybrid of *M. alba* had the highest contents of total phenols and flavonoids, and its four tested antioxidant activities were also the strongest. On the other hand, the hybrid of *B. papyrifera* exhibited the lowest contents of total phenols and flavonoids as well as the four antioxidant activities. Subsequently, through UPLC-QTOF-MS/MS analysis of the five extracts, 23 compounds were identified, of which nine and two compounds were detected for the first time in *Broussonetia* and *Morus* leaves, respectively. Then, 15 phenolics, including seven weak antioxidants and eight strong antioxidants, were screened out by the DPPH/ABTS-guided HPLC method, with compounds 22, 16, 15 being found only in the hybrid of *M. alba*. Combined with structure-activity relationships analysis, we speculated that this may be the major reason for the stronger antioxidant activity of the hybrid of *M. alba*. In order to explore the synergistic or antagonistic effects between the compounds, the interactions of eight strong antioxidants to scavenge DPPH• were further analysed by DPPH-guided HPLC method. Interestingly, the antioxidant activity of flavonoids was inhibited in the mixture, while phenolic acid compounds showed no significant changes. We hypothesised that the stronger antioxidant activity of the hybrid of *M. alba* was mainly contributed by phenolic acid compounds, while phenolic acids have not been identified in the hybrid of *B. papyrifera*, and thus the antioxidant activities were relatively weaker. Nevertheless, the palatability of the hybrids of *B. papyrifera* was better, which may be related to the synthesis mechanism of phenolic compounds. In conclusion, our results contribute greatly to a comprehensive understanding of the potential of the hybrids of *M. alba* and *B. papyrifera* as a source of natural phenolics and antioxidants. This study could also provide useful phytochemical information for them as raw materials for developing functional feeds.

## Supplementary information

Supplementary information

## References

[1] Wang W, Huang B, Qin L (2012). The Genus *Broussonetia*: A Review of its Phytochemistry and Pharmacology. Phytoher. Res..

[2] Ryu HW (2012). Anticholinesterase potential of flavonols from paper mulberry (*Broussonetia papyrifera*) and their kinetic studies. Food Chem..

[3] Baek YS (2009). Tyrosinase inhibitory effects of 1,3-diphenylpropanes from *Broussonetia kazinoki*. Bioorganic Med. Chem..

[4] Han Q (2016). Extraction, antioxidant and antibacterial activities of *Broussonetia papyrifera* fruits polysaccharides. Int. J. Biol. Macromol..

[5] Lee H (2014). The leaves of *Broussonetia kazinoki* siebold inhibit atopic dermatitis-like response on mite allergen-treated Nc/Nga mice. Biomol. Ther..

[6] TSAI F-H (2009). Protective Effect of *Broussonetia papyrifera* against Hydrogen Peroxide-Induced Oxidative Stress in SH-SY5Y Cells. Biosci. Biotechnol. Biochem..

[7] Yang Chunyan, Li Fu, Du Baowen, Chen Bin, Wang Fei, Wang Mingkui (2014). Isolation and Characterization of New Phenolic Compounds with Estrogen Biosynthesis-Inhibiting and Antioxidation Activities from Broussonetia papyrifera Leaves. PLoS ONE.

[8] Ko HH, Chang WL, Lu TM (2008). Antityrosinase and antioxidant effects of ent-kaurane diterpenes from leaves of *Broussonetia papyrifera*. J. Nat. Prod..

[9] Sohn HY, Kwon CS, Son KH (2010). Fungicidal effect of prenylated flavonol, papyriflavonol a, isolated from *Broussonetia papyrifera* (L.) vent. against candida albicans. J. Microbiol. Biotechnol..

[10] Ahn JH (2012). A new pancreatic lipase inhibitor from *Broussonetia kanzinoki*. Bioorganic Med. Chem. Lett..

[11] Dugo P (2009). Characterization of the polyphenolic fraction of *Morus alba* leaves extracts by HPLC coupled to a hybrid IT-TOF MS system. J. Sep. Sci..

[12] Thabti I, Elfalleh W, Hannachi H, Ferchichi A, Campos MDG (2012). Identification and quantification of phenolic acids and flavonol glycosides in Tunisian Morus species by HPLC-DAD and HPLC-MS. J. Funct. Foods.

[13] Gryn-Rynko A, Bazylak G, Olszewska-Slonina D (2016). New potential phytotherapeutics obtained from white mulberry (*Morus alba* L.) leaves. Biomed. Pharmacother..

[14] Riche DM, Riche KD, East HE, Barrett EK, May WL (2017). Impact of mulberry leaf extract on type 2 diabetes (Mul-DM): A randomized, placebo-controlled pilot study. Complement. Ther. Med..

[15] Shim S, Sung S, Lee M (2018). Anti-inflammatory activity of mulberrofuran K isolated from the bark of *Morus bombycis*. Int Immunopharmacol..

[16] Wang, Y., Xiang, L., Wang, C., Tang, C. & He, X. Antidiabetic and Antioxidant Effects and Phytochemicals of Mulberry Fruit (*Morus alba* L.) Polyphenol Enhanced Extract. *PLoS One* **8** (2013). 10.1371/journal.pone.0071144 PMC3728024 23936259

[17] Sánchez-Salcedo EM, Mena P, García-Viguera C, Hernández F, Martínez JJ (2015). (Poly)phenolic compounds and antioxidant activity of white (*Morus alba*) and black (*Morus nigra*) mulberry leaves: Their potential for new products rich in phytochemicals. J. Funct. Foods.

[18] Park E, Lee SM, Lee J (2013). eun & Kim, J. H. Anti-inflammatory activity of mulberry leaf extract through inhibition of NF-κB. J. Funct. Foods.

[19] Li H (2015). Separation of vitexin-4″-O-glucoside and vitexin-2″-O-rhamnoside from hawthorn leaves extracts using macroporous resins. J. Chromatogr. B Anal. Technol. Biomed. Life Sci..

[20] Yang L (2018). Seasonal dynamics of constitutive levels of phenolic components lead to alterations of antioxidant capacities in *Acer truncatum* leaves. Arab. J. Chem..

[21] Meda NR (2017). Characterization of antioxidants from *Detarium microcarpum* Guill. et Perr. leaves using HPLC-DAD coupled with pre-column DPPH assay. Eur. Food Res. Technol..

[22] Zhu J, Yi X, Zhang J, Chen S, Wu Y (2017). Chemical profiling and antioxidant evaluation of Yangxinshi Tablet by HPLC–ESI-Q-TOF-MS/MS combined with DPPH assay. J. Chromatogr. B Anal. Technol. Biomed. Life Sci..

[23] Zhang C, Shen X, Chen J, Jiang X, Hu FL (2017). Identification of Free Radical Scavengers from Brazilian Green Propolis Using Off-Line HPLC-DPPH Assay and LC-MS. J. Food Sci..

[24] Qiu J (2012). Screening natural antioxidants in peanut shell using DPPH-HPLC-DAD-TOF/MS methods. Food Chem..

[25] Kim D-S (2014). Antioxidant activities and polyphenol content of *Morus alba* leaf extracts collected from varying regions. Biomed. Reports.

[26] Mahmoud AM, Abd El-Twab SM, Abdel-Reheim ES (2017). Consumption of polyphenol-rich Morus alba leaves extract attenuates early diabetic retinopathy: the underlying mechanism. Eur. J. Nutr..

[27] Wang W, Zu Y, Fu Y, Efferth T (2012). *In Vitro* Antioxidant and Antimicrobial Activity of Extracts from *Morus alba* L. Leaves, Stems and Fruits. Am. J. Chin. Med..

[28] Radojković M, Moreira MM, Soares CM, Barroso F (2018). Microwave-assisted extraction of phenolic compounds from *Morus nigra* leaves: optimization and characterization of the antioxidant activity and phenolic composition. J Chem Technol Biot..

[29] Fu Q (2016). Rapid screening and identification of compounds with DNA-binding activity from Folium Citri Reticulatae using on-line HPLC-DAD-MS^n^ coupled with a post column fluorescence detection system. Food Chem..

[30] Melguizo-Melguizo D, Diaz-de-Cerio E, Quirantes-Piné R, Švarc-Gajić J, Segura-Carretero A (2014). The potential of *Artemisia vulgaris* leaves as a source of antioxidant phenolic compounds. J. Funct. Foods.

[31] Clifford MN, Johnston KL, Knight S, Kuhnert N (2003). Hierarchical scheme for LC-MSn identification of chlorogenic acids. J. Agric. Food Chem..

[32] Crupi P (2018). Comprehensive identification and quantification of chlorogenic acids in sweet cherry by tandem mass spectrometry techniques. J. Food Compos. Anal..

[33] Jaiswal R, Sovdat T, Vivan F, Kuhnert N (2010). Profiling and characterization by LC-MSnof the chlorogenic acids and hydroxycinnamoylshikimate esters in maté (Ilex paraguariensis). J. Agric. Food Chem..

[34] Benayad Z, Gómez-Cordovés C, Es-Safi NE (2014). Identification and quantification of flavonoid glycosides from fenugreek (*Trigonella foenum-graecum*) germinated seeds by LC-DAD-ESI/MS analysis. J. Food Compos. Anal..

[35] Feng W (2008). Chemical constituents from the leaves of Brousson papyrifera. Acta Pharm Sin..

[36] Zhao Y, Wang Y, Jiang ZT, Li R (2017). Screening and evaluation of active compounds in polyphenol mixtures by HPLC coupled with chemical methodology and its application. Food Chem..

[37] Tang D, Li HJ, Chen J, Guo CW, Li P (2008). Rapid and simple method for screening of natural antioxidants from Chinese herb Flos Lonicerae Japonicae by DPPH-HPLC-DAD-TOF/MS. J. Sep. Sci..

[38] Baranowska I, Bajkacz S (2018). A new UHPLC-MS/MS method for the determination of flavonoids in supplements and DPPH[rad]-UHPLC-UV method for the evaluation of the radical scavenging activity of flavonoids. Food Chem..

[39] Hu X (2016). Antioxidant capacity and phenolic compounds of *Lonicerae macranthoides* by HPLC-DAD-QTOF-MS/MS. J. Pharm. Biomed. Anal..

[40] Senthil Kumar K, Kumaresan R (2013). Theoretical investigation of the conformational, electronic and antioxidant properties of azaleatin, isorhamnetin and quercetagetin. Mol. Simul..

[41] Seyoum A, Asres K, El-Fiky FK (2006). Structure-radical scavenging activity relationships of flavonoids. Phytochemistry.

[42] Burda S, Oleszek W (2001). Antioxidant and antiradical activities of flavonoids. J. Agric. Food Chem..

[43] Cai (2006). scavenging activity relationships of phenolic compounds from traditional Chinese medicinal plants. Life Sci..

[44] Kelly EH, Dennis JB, Anthony RT (2002). Flavonoid antioxidants: chemistry, metabolism and structure-activity relationships. J. Nutr. Biochem..

[45] Yu M (2019). Comparison of free, esterified, and insoluble-bound phenolics and their bioactivities in three organs of *Lonicera japonica* and *L. macranthoides*. Molecules.

[46] Fan H (2018). Assessment of the bioactive phenolic composition of *Acer truncatum* seed coat as a byproduct of seed oil. Ind. Crops Prod..

[47] Yin P (2019). Bioactive components and antioxidant activities of oak cup crude extract and its four partially purified fractions by HPD-100 macroporous resin chromatography. Arab. J. Chem..

[48] Li K (2018). Structure-activity relationship of eight high content flavonoids analyzed with a preliminary assign-score method and their contribution to antioxidant ability of flavonoids-rich extract from *Scutellaria baicalensis* shoots. Arab. J. Chem..

[49] Wang L, Luo Y, Wu Y, Xia F, Wu Z (2018). Quickly verifying the antioxidant contribution of the individual composition in natural antioxidants by HPLC-free radical scavenging detection. Lwt.

