# Supplementary material for: Antioxidant evaluation-guided chemical profiling and structure-activity analysis of leaf extracts from five trees in Broussonetia and Morus (Moraceae)
Source: Sci Rep. 2020 Mar 16;10:4808. doi: 10.1038/s41598-020-61709-5 (PMC7075987; doi:10.1038/s41598-020-61709-5)
Supplement: Supplementary file 1 — Supplementary information [file 41598_2020_61709_MOESM1_ESM.pdf]

Antioxidant evaluation-guided chemical profiling and structure-activity analysis of leaf extracts from five trees in *Broussonetia* and *Morus* (Moraceae)

Xinxin Cao[1], Lingguang Yang[1], Qiang Xue[1], Fan Yao[1], Jing Sun[1], Fuyu Yang[2], and Yujun Liu[1,*]

Supplementary Figures

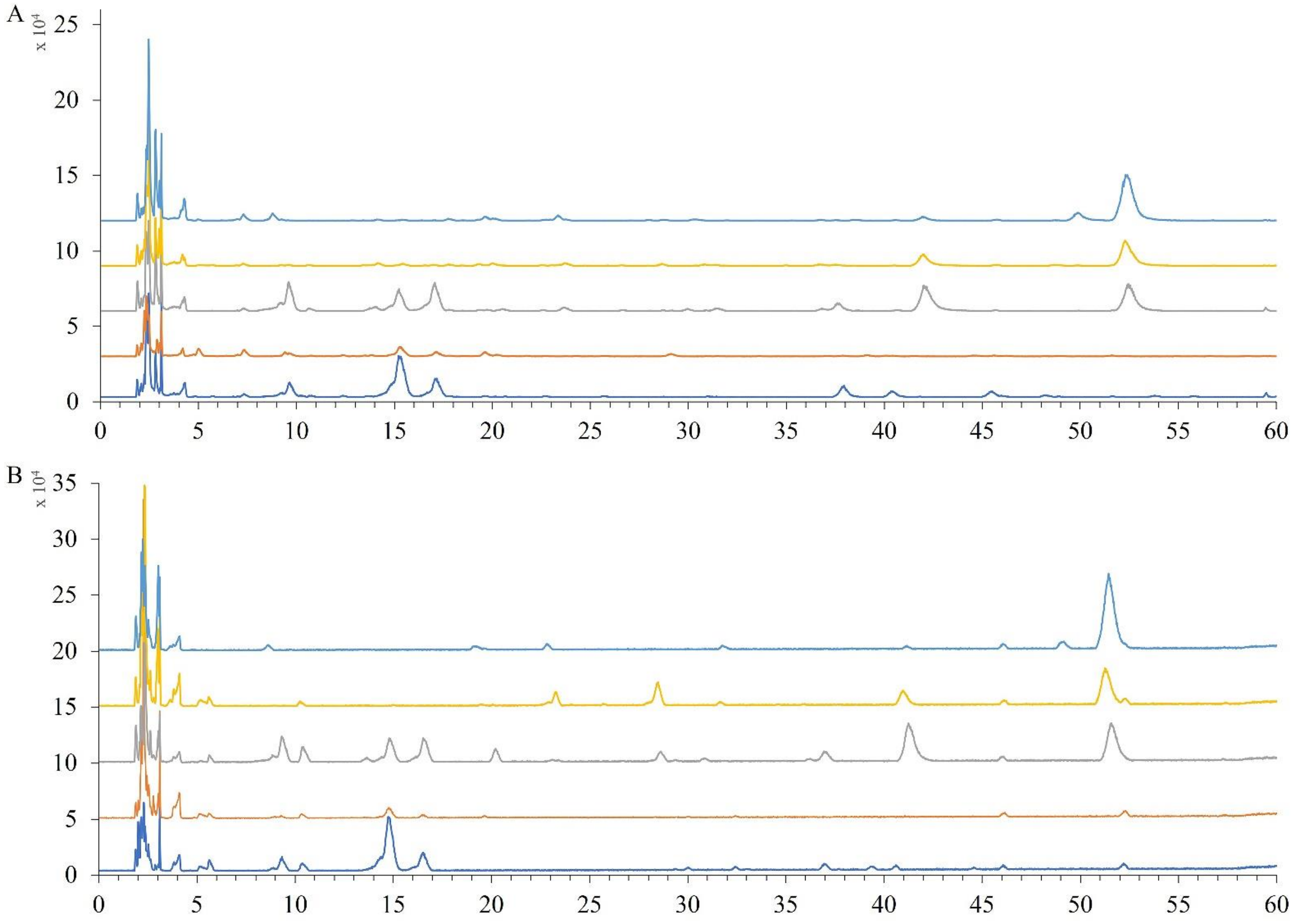

Figure S1. Ultra-performance liquid chromatography–quadrupole time-of-flight tandem mass-spectroscopy (UPLC–QTOF–MS/MS) profiles of the five leaf extracts obtained under optimized conditions (from top to bottom, HBP, BP, BK, MA and HMA). (A) Negative ion mode; (B) positive ion mode.

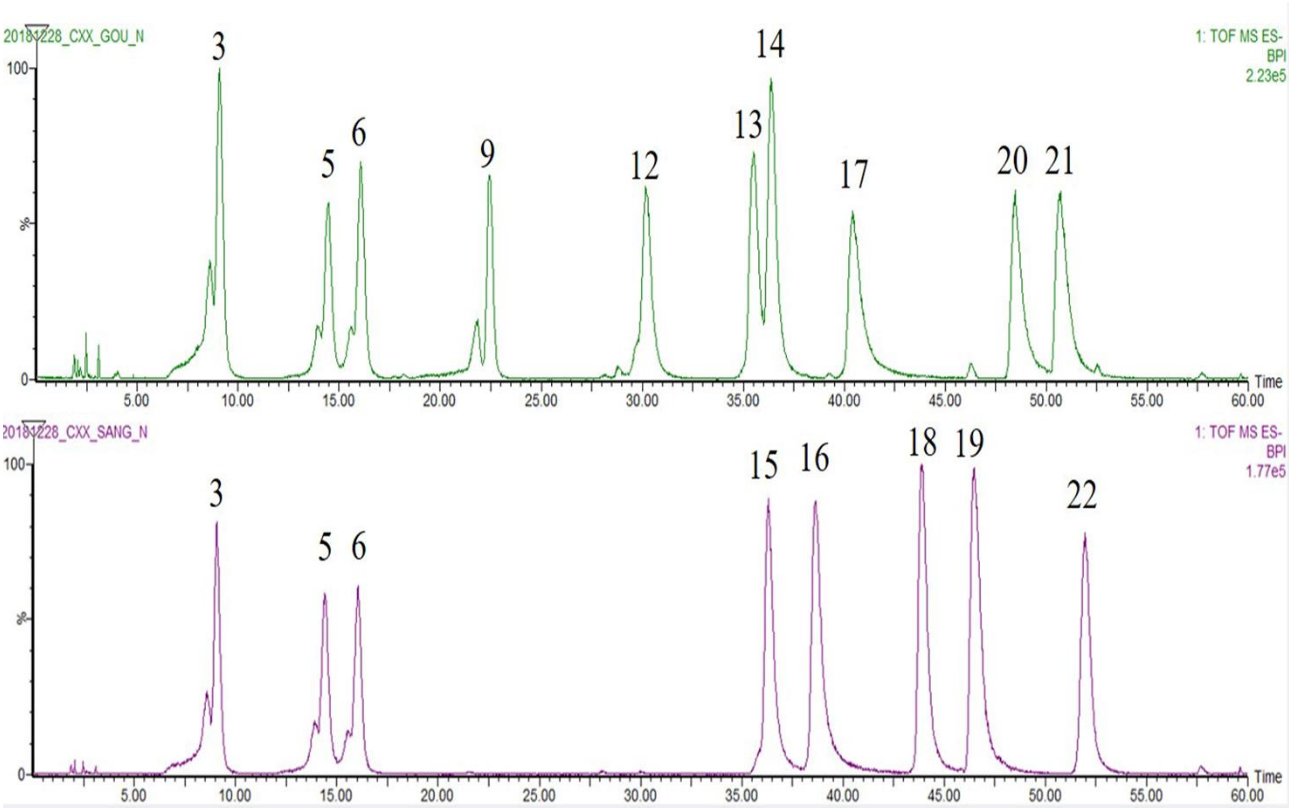

Figure S2. UPLC–QTOF–MS/MS profiles of the 15 authentic standards obtained under optimized conditions. Peak numbers for individual phenolic compounds are as listed in Tables 1 and 2: 5-caffeoylquinic acid (3); 3-caffeoylquinic acid (5); 4-caffeoylquinic acid (6); vicenin-2 (9); orientin (12); vitexin (13); isovitexin (14); rutin (15); isoquercitrin (16); luteolin-7-*O*-glucuronide (17); kaempferol-3-*O*-rutinoside (18); kaempferol-3-*O*-glucoside (19); apigenin 7-*O*-glucoside (20); apigenin-7-*O*-glucuronide (21); 4,5-dicaffeoylquinic acid (22).

Supplementary Table

Table S1. Peak areas of extracts.

Species	Peak	Analytes	PK0	PK1	PK5	PK10	PK7
MA	3	5-Caffeoylquinic acid	409,340	131,177	64,208	36,978	189,506
	5	3-Caffeoylquinic acid	1,140,593	377,907	95,994	60,574	573,742
	6	4-Caffeoylquinic acid	384,022	107,506	79,719	64,160	173,119
	15	Rutin	58,590	9,327.667	7403.333	5,807.333	14,424.667
	16	Isoquercitrin	58,114	7,039.333	0	0	11,132
	19	Kaempferol-3-*O*-glucoside	37,454	4012	11097.667	2,306.667	–*
HMA	3	5-Caffeoylquinic acid	2,256,111	673,441	294,759	73,533	724,905
	5	3-Caffeoylquinic acid	9,759,604	3,392,971	968,466	291,202	3,593,089
	6	4-Caffeoylquinic acid	3,018,856	949,305	325,096	80,969	993,362
	15	Rutin	771,584	236,064	10,356	9,472	277,638
	16	Isoquercitrin	559,658	115,938	6,061	0	128,383
	18	Kaempferol-3-*O*-rutinoside	628,571	267,140	151,664	119,551	534,455
	19	Kaempferol-3-*O*-glucoside	405,373	106,294	46,145	30,479	215,197
	22	4,5-Dicaffeoylquinic acid	375,113	95,081	10,562	7,845	112,132
BP	3	5-Caffeoylquinic acid	257,348	31,929	13,589	15,842	61,962
	6	4-Caffeoylquinic acid	331,144	53,634	23,245	23,234	71,296
	12	Orientin	149,969	1,658	0	0	25,589
	13	Vitexin	197,528	46,739	44,905	44,705	48,775
	14	Isovitexin	226,861	56,535	50,710	50,307	57,394
	17	Luteolin-7-*O*-glucuronide	1,871,251	29,462	0	0	403,495
	21	Apigenin-7-*O*-glucuronide	4,943,131	1,497,898	1,437,140	1,410,739	1,509,926
HBP	9	Vicenin-2	571,047	214,156	205,851	198,704	216,117
	13	Vitexin	74,102	22,123	22,637	21,686	23,875
	14	Isovitexin	107,615	34,635	32,687	30,528	34,839
	17	Luteolin-7-*O*-glucuronide	441,761	0	0	0	104,718
	20	Apigenin 7-glucoside	966,068	314,247	308,513	287,503	349,096
	21	Apigenin-7-*O*-glucruonide	9,492,549	3,442,524	3,272,994	3,024,951	3,486,014
BK	3	5-Caffeoylquinic acid	4,068,748	1,341,633	435,516	130,898	1,641,436
	5	3-Caffeoylquinic acid	3,934,672	1,085,867	393,011	147,641	1,370,160
	6	4-Caffeoylquinic acid	4,853,944	1,141,226	436,414	153,044	1,468,388
	12	Orientin	307,739	82,763	1,995	0	99,037
	14	Isovitexin	1,138,799	365,801	309,339	257,594	384,207
	17	Luteolin-7-*O*-glucuronide	4,494,512	1,250,890	31,990	2,667	1,398,513
	21	Apigenin-7-*O*-glucronide	5,120,042	1,581,017	1,414,865	1,650,471	1,678,148

PK0: initial peak area of samples before reaction with free radicals. PK1, PK5, PK10, and PK7: final peak areas of extracts after reaction with 1, 5, and 10 mM DPPH and 7 mM ABTS, respectively. Peak areas are means of three independent experiments (n = 3), standard data not shown. –*: signal covered by the ABTS peak.
